# Beyond Weight Loss: Gut Microenvironment Modulation to Enhance Cardiometabolic Outcomes and Long-Term Adherence During Incretin-Based Therapy

**DOI:** 10.3390/jcm15155909

**Published:** 2026-07-29

**Authors:** Calogero Geraci, Francesca La Rocca, Salvatore Massimo Petrina, Agostino Buonauro, Valentina Morello, Valentina Paternò, Ciro Santoro, Giulio Geraci, Roberta Esposito

**Affiliations:** 1 Cardio Obesity Group, ACRIS APS (Associazione Cuore, Reni e Ipertensione Sicilia), 93100 San Cataldo, CL, Italy; 2 Cardiology Unit, Azienda Sanitaria Provinciale of Caltanissetta (ASP), 93100 Caltanissetta, CL, Italy; 3 Department of Clinical and Experimental Medicine, University of Catania, 95122 Catania, CT, Italy; francescalarocca9401@gmail.com; 4 Cardiology Unit, Azienda Sanitaria Provinciale of Ragusa (ASP), 97100 Ragusa, RG, Italy; 5 Santa Maria Della Pietà Hospital, ASL Napoli 3 Sud, 80035 Nola, NA, Italy; 6 Community Nutrition Biologist, 93100 Caltanissetta, CL, Italy; 7 Department of Medicine and Surgery, Kore University of Enna, 94100 Enna, EN, Italy; 8 Department of Pharmacy Health and Nutritional Science, Univertisy of Calabria Rende (CS), 87036 Arcavacata, CS, Italy; 9 Department of Clinical Medicine and Surgery, Federico II University Hospital, 80131 Naples, NA, Italy

**Keywords:** GLP-1 receptor agonists, dual GIP/GLP-1 receptor agonists, obesity, gut microbiota, gastrointestinal tolerability, treatment adherence, short-chain fatty acids, partially hydrolyzed guar gum, cardiometabolic disease, gut–heart axis

## Abstract

**Background:** Glucagon-like peptide-1 receptor agonists and dual glucose-dependent insulinotropic polypeptide/glucagon-like peptide-1 (GIP/GLP-1) receptor agonists have substantially improved the management of obesity and cardiometabolic disease, providing significant benefits on weight reduction, glycemic control, and cardiovascular outcomes. Despite these advances, gastrointestinal (GI) adverse events remain a major cause of dose reduction, treatment interruption, and poor long-term adherence. Increasing evidence suggests that interactions between incretin-based therapies and the intestinal microenvironment may contribute to GI tolerability and influence treatment persistence. **Methods:** We conducted a narrative review of the literature examining the relationship between incretin-based therapies, gut microbiota, intestinal barrier function, and microbial metabolites. Evidence from randomized clinical trials, observational studies, systematic reviews, and mechanistic investigations was evaluated to explore potential gut-directed supportive strategies during incretin-based treatment. **Results:** Preclinical and mechanistic evidence suggests possible bidirectional interactions between incretin pharmacology and the intestinal microenvironment, whereas direct human evidence remains limited and inconsistent. Alterations in gastrointestinal motility induced by GLP-1 receptor agonists could theoretically influence microbial composition and fermentation dynamics, although human studies have not consistently demonstrated significant microbiota changes. Based on these observations, we propose the Incretin–Microbiota Tolerance Axis (IMTA) as a conceptual framework linking gut homeostasis, GI tolerability, and treatment adherence. Among potential supportive interventions, partially hydrolyzed guar gum (PHGG) may promote SCFA-producing microbiota and improve bowel function, whereas simethicone may provide symptomatic relief of gas-related discomfort during dose escalation. SCFA-supportive nutritional approaches may further contribute to maintenance of intestinal homeostasis. **Conclusions:** Optimization of the intestinal microenvironment may represent a complementary strategy to improve GI tolerability and support long-term adherence during incretin-based therapy. Although the IMTA framework is supported by biological plausibility and emerging evidence, prospective clinical studies are required to determine whether microbiota-targeted interventions improve treatment persistence and cardiometabolic outcomes in patients receiving GLP-1 receptor agonists or dual GIP/GLP-1 receptor agonists.

## 1. Introduction

Glucagon-like peptide-1 receptor agonists and dual GIP/GLP-1 receptor agonists have substantially reshaped the therapeutic landscape of obesity and cardiometabolic disease. In the STEP 1 trial, semaglutide 2.4 mg weekly achieved a mean body weight reduction of 14.9% compared with 2.4% with placebo [1], whereas tirzepatide 15 mg weekly produced weight reductions approaching 21% in SURMOUNT-1 [2], establishing new benchmarks for pharmacological obesity management. Importantly, semaglutide also demonstrated a significant reduction in major adverse cardiovascular events (MACE) among overweight or obese individuals without diabetes in the SELECT trial [3], supporting the concept that incretin-based therapies may provide cardiovascular benefit beyond weight reduction alone.

Despite these advances, a substantial gap persists between efficacy observed in randomized clinical trials and effectiveness achieved in routine clinical practice. Recent real-world evidence suggests that approximately 46–65% of patients discontinue GLP-1 receptor agonist or dual GIP/GLP-1 receptor agonist therapy within the first year. Treatment discontinuation is multifactorial and may reflect gastrointestinal adverse events together with medication cost, healthcare access, drug availability, patient preference, perceived treatment effectiveness, and comorbidities. Nevertheless, gastrointestinal intolerance remains one of the most frequent clinically modifiable contributors to early treatment interruption. Commonly reported symptoms include nausea, constipation, bloating, vomiting, and early satiety [4]. Premature treatment interruption may limit not only sustained weight reduction but also the long-term metabolic and cardiovascular benefits associated with continued incretin therapy, as suggested by the weight regain and deterioration of several cardiometabolic risk markers observed after semaglutide withdrawal in the STEP 1 extension study [4,5].

It has been hypothesized that the intestinal microenvironment may contribute to interindividual differences in gastrointestinal tolerability; however, direct human evidence supporting this relationship during incretin therapy remains limited. GLP-1 and GIP receptors are widely expressed throughout the enteric nervous system, gastrointestinal mucosa, and myenteric plexus, where receptor activation modulates gastric emptying, intestinal motility, nutrient transit, and luminal fermentation dynamics. These pharmacologically induced changes may interact with the gut microbiota, although the magnitude and clinical relevance of this interaction in humans remain uncertain [6,7].

A dysbiotic gut environment has been proposed as a potential modifier of gastrointestinal tolerability, but this hypothesis has not yet been prospectively validated in incretin-treated patients [8,9]. Conversely, preservation of a metabolically active, SCFA-producing microbiota may support epithelial barrier integrity, attenuate GI adverse events, and potentially enhance endogenous incretin signaling through gut–incretin interactions [8,9].

In this context, we propose the concept of the Incretin–Microbiota Tolerance Axis (IMTA), a mechanistically oriented framework suggesting that optimization of the gut microenvironment may represent an important supportive component of incretin-based cardiometabolic therapy. This review examines the rationale supporting three complementary and clinically accessible strategies—partially hydrolyzed guar gum (PHGG), simethicone, and SCFA-supportive interventions—that may improve GI tolerability, facilitate long-term adherence, and contribute to broader cardiometabolic benefit during incretin-based treatment.

The present review does not aim to provide a comprehensive overview of all supportive strategies currently recommended during incretin therapy. Instead, it focuses on three representative interventions selected because they target complementary domains of the proposed IMTA framework: (i) PHGG as a microbiota-supportive prebiotic intervention with potential effects on gastrointestinal motility and SCFA production; (ii) simethicone as a symptomatic strategy targeting luminal gas-related discomfort; and (iii) SCFA-supportive nutritional approaches as modulators of microbial metabolic function. Other clinically relevant supportive measures—including individualized dose titration, dietary counseling, hydration, antiemetic therapy, constipation-directed treatments, resistance exercise, and nutritional optimization—remain essential components of standard clinical care but fall outside the specific mechanistic scope of this conceptual framework.

### Core Thesis: The Incretin–Microbiota Tolerance Axis (IMTA)

The long-term cardiometabolic benefits of incretin-based therapy depend on sustained treatment adherence. In clinical practice, adherence is frequently limited by gastrointestinal tolerability. Increasing evidence suggests that GI tolerability may be influenced by multiple patient-, treatment-, and gastrointestinal factors. The functional status of the intestinal microenvironment is proposed as one potential modifier, but its independent contribution remains uncertain.

Accordingly, modulation of the gut microenvironment may represent more than a supportive intervention during incretin therapy. Within the proposed IMTA framework, preservation of intestinal homeostasis is explored as a potential supportive factor of treatment persistence, therapeutic tolerability, and long-term cardiometabolic outcomes.

**Figure 1:**
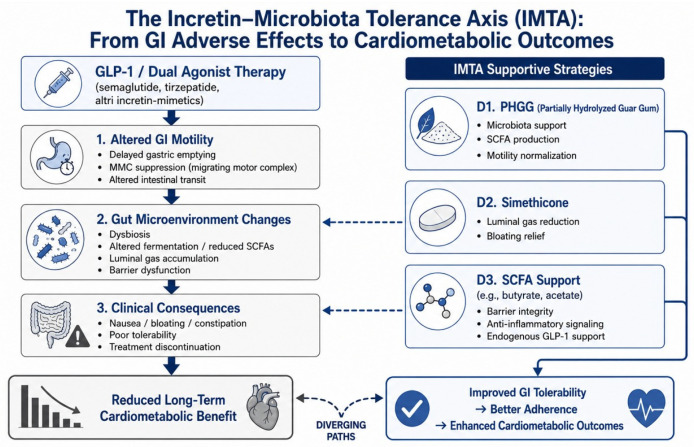
The proposed Incretin–Microbiota Tolerance Axis (IMTA). Hypothesis-generating framework illustrating separately supported and putative relationships among incretin-induced gastrointestinal effects, possible changes in the intestinal microenvironment, gastrointestinal symptom burden, treatment persistence, and cardiometabolic outcomes. Solid arrows indicate clinically established or relatively well-supported associations, whereas dashed arrows indicate hypothetical or insufficiently validated links. The figure should not be interpreted as demonstrating a proven causal sequence.

To summarize the proposed conceptual framework, Figure 1 illustrates the Incretin–Microbiota Tolerance Axis (IMTA), highlighting the interplay between incretin-induced gastrointestinal changes, gut microenvironment alterations, treatment adherence, and cardiometabolic outcomes.

## 2. Methods

This narrative review aimed to summarize and critically discuss the current evidence regarding the relationships between incretin-based therapies, gastrointestinal tolerability, gut microbiota, microbial metabolites, treatment adherence, and cardiometabolic outcomes.

A literature search was performed in PubMed/MEDLINE, Scopus, and Google Scholar up to July 2026 using combinations of the following keywords: “GLP-1 receptor agonists”, “dual GIP/GLP-1 receptor agonists”, “semaglutide”, “tirzepatide”, “gut microbiota”, “microbiome”, “short-chain fatty acids”, “SCFA”, “partially hydrolyzed guar gum”, “PHGG”, “simethicone”, “gastrointestinal adverse events”, “treatment adherence”, “obesity”, “cardiometabolic outcomes”, and “gut–heart axis”.

Priority was given to randomized controlled trials, systematic reviews, meta-analyses, large observational studies, and high-quality mechanistic investigations published in peer-reviewed journals. Additional relevant publications were identified through manual screening of the reference lists of selected articles.

Given the heterogeneous and predominantly mechanistic nature of the available literature, a formal systematic review or meta-analysis was not considered appropriate. Instead, the retrieved evidence was narratively synthesized to develop the hypothesis-generating conceptual framework presented in this review.

Clinical recommendations discussed throughout the manuscript should therefore be interpreted as hypothesis-generating supportive considerations rather than evidence-based therapeutic recommendations.

During the preparation of this manuscript, the authors used ChatGPT (OpenAI, https://chatgpt.com) to assist with language editing, improvement of readability, and revision of the manuscript in response to peer-review comments. All scientific content, interpretation of the literature, conclusions, and final editorial decisions were critically reviewed and approved by the authors, who take full responsibility for the content of the manuscript.

## 3. The Weight-Loss Paradox: Efficacy Undermined by Tolerability

### 3.1. Magnitude of the Adherence Problem

The clinical development programs for semaglutide and tirzepatide demonstrated substantial efficacy in obesity management; however, treatment persistence in real-world practice remains considerably lower than that observed in randomized clinical trials. Within controlled trial settings, gastrointestinal (GI) adverse events are typically mitigated through structured dose-escalation protocols, close clinical monitoring, and standardized supportive care. In the STEP 1 trial, discontinuation due to GI adverse events occurred in 4.5% of semaglutide-treated participants [1], whereas SURMOUNT-1 reported discontinuation rates of approximately 4.3% among patients receiving tirzepatide 15 mg weekly [2].

In routine clinical practice, however, treatment interruption appears substantially more frequent. Real-world analyses suggest that 46–65% of patients discontinue GLP-1 receptor agonist or dual GIP/GLP-1 receptor agonist therapy within the first year. Although gastrointestinal symptoms are among the most frequently reported reasons for discontinuation, persistence is influenced by multiple clinical and non-clinical factors, including treatment cost, medication availability, healthcare access, patient preference, perceived effectiveness, and comorbidities [4]. This discrepancy highlights the gap between efficacy under trial conditions and long-term effectiveness in broader patient populations.

The clinical implications of reduced adherence extend beyond weight-loss maintenance alone. In the SELECT trial, semaglutide administered over a median follow-up period of approximately 34 months was associated with a significant reduction in major adverse cardiovascular events [3,4]. Because these benefits are likely exposure-dependent, premature treatment discontinuation may limit not only sustained metabolic improvement but also the long-term cardiovascular protection associated with continued incretin therapy.

Accordingly, impaired treatment persistence may represent one of the principal limitations of incretin-based pharmacotherapy in contemporary obesity management. Better understanding the mechanisms underlying GI intolerance—including the potential contribution of altered intestinal motility, dysbiosis, and impaired gut barrier function—may therefore be clinically relevant to improving adherence and optimizing long-term therapeutic outcomes.

### 3.2. Potential Cardiometabolic Consequences of Gastrointestinal Intolerance and Treatment Interruption

Beyond their impact on treatment adherence, gastrointestinal intolerance during incretin therapy may indirectly influence cardiometabolic health through several interconnected mechanisms, including changes in dietary intake, intestinal physiology, and treatment persistence. However, gastrointestinal symptoms themselves are not established cardiovascular risk factors. Persistent nausea, reduced appetite, and early satiety can adversely affect dietary quality and nutritional adequacy in susceptible individuals. In addition, constipation and altered intestinal transit may contribute to unfavorable shifts in gut microbial composition, including reduced abundance of short-chain fatty acid (SCFA)-producing taxa and altered fermentation dynamics [6,7,8,9].

These alterations have been hypothesized to contribute to impaired intestinal barrier integrity, increased lipopolysaccharide (LPS) translocation, and low-grade systemic inflammation, processes increasingly implicated in insulin resistance, endothelial dysfunction, and atherosclerotic disease progression [7,8]. Although direct causal relationships remain incompletely established in patients receiving incretin-based therapy, this mechanistic framework provides a biologically plausible link between GI dysfunction, intestinal dysbiosis, and residual cardiometabolic risk. These mechanisms remain biologically plausible but have not been directly demonstrated in patients receiving incretin-based therapies.

Importantly, any potential cardiometabolic consequence should be interpreted as deriving primarily from treatment interruption and loss of exposure to effective therapy rather than from gastrointestinal symptoms per se [5]. From this perspective, interventions aimed at preserving intestinal homeostasis during incretin therapy may have implications extending beyond symptomatic relief by supporting treatment persistence and, indirectly, the maintenance of long-term metabolic and cardiovascular benefit.

The STEP 1 extension demonstrated that discontinuation of semaglutide was associated with substantial weight regain and deterioration of several cardiometabolic risk markers following treatment withdrawal [4]. Conversely, the SELECT trial demonstrated cardiovascular benefit during continued semaglutide treatment [3]. Together, these studies support the importance of treatment persistence but do not establish that gastrointestinal intolerance or microbiota alterations directly mediate cardiovascular outcomes. More recently, GLP-1 receptor agonists and dual GIP/GLP-1 receptor agonists have also been associated with a reduced incidence of atrial fibrillation, although the underlying biological mechanisms remain unresolved [10].

### 3.3. GLP-1/GIP Receptor Distribution and Enteric Pharmacology

GLP-1 receptors are widely expressed throughout the gastrointestinal tract, including on vagal afferent neurons, enteroendocrine L-cells, enteric neurons, intestinal smooth muscle cells, and epithelial cells. Pharmacological receptor activation influences multiple aspects of gastrointestinal physiology, including gastric emptying, pyloric tone, intestinal motility, gastric acid secretion, and postprandial transit dynamics. These effects contribute substantially to both the therapeutic efficacy and GI adverse event profile of incretin-based therapies.

Dual GIP/GLP-1 receptor agonism further increases the complexity of enteric pharmacodynamics. Compared with semaglutide, tirzepatide may exhibit a somewhat distinct GI tolerability profile, potentially reflecting differences in receptor kinetics, gastric accommodation, or GIP-mediated modulation of intestinal motility [2]. Nevertheless, as dose escalation progresses, both agents are commonly associated with nausea, bloating, constipation, and altered bowel habits that frequently require active clinical management.

Importantly, these motility-related alterations also modify the luminal environment in which microbial fermentation occurs. Delayed transit and altered nutrient delivery may influence microbial composition, gas production, and SCFA generation, thereby creating a bidirectional interaction between incretin pharmacology and the intestinal microenvironment [6,7]. This interaction represents a central mechanistic basis for the proposed Incretin–Microbiota Tolerance Axis framework.

### 3.4. Human Evidence Linking Incretin Therapy and Gut Microbiota

Experimental and translational studies suggest that incretin-based therapies may influence the intestinal microenvironment through changes in gastrointestinal motility, nutrient delivery, body weight, and metabolic status. However, evidence from human studies remains limited and inconsistent. Experimental observations therefore cannot be directly extrapolated to clinical populations receiving GLP-1 receptor agonists or dual GIP/GLP-1 receptor agonists [6,7].

In a randomized controlled trial involving 51 individuals with type 2 diabetes, Smits et al. found no significant changes in gut microbial alpha diversity, beta diversity, or overall taxonomic composition following liraglutide treatment, despite clear metabolic improvements. These findings suggest that the clinical effects of liraglutide are unlikely to depend on a consistent restructuring of the gut microbiota [8].

Similarly, Klemets et al. recently evaluated longitudinal microbiome changes in semaglutide-treated patients and found no statistically significant alterations in overall microbial diversity or composition after correction for multiple testing. Although exploratory analyses suggested that baseline microbiota characteristics might be associated with glycemic response, these observations require confirmation in larger prospective cohorts [9].

Overall, current human evidence does not support a reproducible microbiota-modifying effect of incretin therapy. Consequently, within the proposed Incretin–Microbiota Tolerance Axis (IMTA), alterations of the gut microbiota should be regarded as a biologically plausible but unproven intermediate mechanism, warranting further investigation in adequately powered prospective studies.

Recent reviews have further emphasized the importance of the intestinal microenvironment, epithelial barrier integrity, and host–microbiota interactions in systemic metabolic diseases. These observations reinforce the biological plausibility of gut-mediated mechanisms but do not provide direct evidence that microbiota modulation improves gastrointestinal tolerability or treatment persistence during incretin-based therapy [11].

## 4. Partially Hydrolyzed Guar Gum (PHGG): Prebiotic Microbiota Support and Motility Normalization

### 4.1. Rationale and Biological Properties

Partially hydrolyzed guar gum (PHGG) is a soluble, non-viscous dietary fiber obtained through enzymatic hydrolysis of guar gum. Compared with native guar gum and other highly fermentable fibers, PHGG exhibits lower viscosity while retaining prebiotic properties, resulting in generally favorable gastrointestinal tolerability. These characteristics have generated interest in PHGG as a potential supportive intervention in clinical settings characterized by altered intestinal transit and microbiota imbalance [12,13].

PHGG reaches the colon largely undigested, where it undergoes bacterial fermentation and may promote the growth of beneficial microbial taxa, including *Bifidobacterium* spp. and *Faecalibacterium prausnitzii*, organisms associated with short-chain fatty acid (SCFA) production and intestinal homeostasis [12,13,14,15]. Through these mechanisms, PHGG has been proposed as a potential modulator of the intestinal microenvironment.

### 4.2. Evidence from Functional Gastrointestinal Disorders

Most clinical evidence supporting PHGG derives from studies conducted in patients with functional gastrointestinal disorders rather than in individuals receiving incretin-based therapies. In randomized and observational studies involving irritable bowel syndrome (IBS), PHGG supplementation has been associated with improvements in stool consistency, bowel habit regulation, and overall gastrointestinal symptom burden [13,14,15].

These findings suggest that PHGG may contribute to normalization of intestinal transit and modulation of microbial fermentation patterns. However, extrapolation of these observations to patients receiving GLP-1 receptor agonists or dual GIP/GLP-1 receptor agonists should be approached with caution, as the mechanisms underlying GI symptoms during incretin therapy may differ from those observed in functional bowel disorders.

### 4.3. Potential Relevance to the IMTA Framework

Within the proposed Incretin–Microbiota Tolerance Axis (IMTA), PHGG may represent a candidate strategy for supporting microbial metabolic activity and maintaining intestinal homeostasis. Experimental and translational studies suggest that PHGG fermentation may increase SCFA availability, particularly butyrate, which contributes to epithelial barrier integrity and modulation of mucosal inflammatory responses [7,12,13,14,15,16].

Because GLP-1 receptor agonists alter gastrointestinal motility and may influence fermentation dynamics, preservation of SCFA-producing microbiota could theoretically facilitate gastrointestinal adaptation during treatment. Nevertheless, direct evidence supporting this hypothesis remains limited.

Importantly, no randomized clinical trials have specifically evaluated PHGG supplementation in patients receiving semaglutide, tirzepatide, or other incretin-based therapies. Therefore, any potential benefits on gastrointestinal tolerability, treatment adherence, or cardiometabolic outcomes remain speculative and require prospective validation.

### 4.4. Current Clinical Perspective

Given its favorable safety profile and established use in functional gastrointestinal disorders, PHGG may be considered a low-risk nutritional adjunct in selected patients experiencing constipation-predominant symptoms or low dietary fiber intake during incretin therapy. However, current evidence is insufficient to support routine use as a standard adjunctive intervention during GLP-1 receptor agonist or dual GIP/GLP-1 receptor agonist treatment.

Future studies should determine whether PHGG supplementation improves gastrointestinal symptom burden, treatment persistence, and cardiometabolic outcomes during incretin-based therapy.

## 5. Simethicone: Symptom Relief Within the Luminal Gas Regulation Domain of IMTA

### 5.1. Mechanism of Action and Pharmacological Profile

Simethicone is a non-systemic anti-foaming agent composed of polydimethylsiloxane and silicon dioxide that acts exclusively within the gastrointestinal lumen. By reducing the surface tension of gas bubbles, simethicone promotes their coalescence into larger pockets that can be more easily eliminated through belching or flatulence. Because it is neither absorbed nor metabolized, simethicone does not exert direct effects on gastrointestinal motility, nutrient absorption, intestinal permeability, or gut microbiota composition.

This pharmacological profile distinguishes simethicone from microbiota-directed interventions such as dietary fiber supplementation or SCFA-supportive strategies. Rather than modifying the underlying intestinal microenvironment, simethicone primarily provides symptomatic relief from gas-related discomfort.

### 5.2. Gas Accumulation During Incretin Therapy

GLP-1 receptor agonists and dual GIP/GLP-1 receptor agonists substantially alter gastrointestinal motility through delayed gastric emptying and changes in intestinal transit. These effects may promote retention of fermentable substrates and contribute to luminal gas accumulation, particularly during treatment initiation and dose escalation.

Clinically, patients frequently report bloating, abdominal distension, postprandial fullness, and excessive belching among the most bothersome gastrointestinal adverse events associated with incretin therapy. Although these symptoms are generally transient, they may negatively affect treatment satisfaction and contribute to early dose reduction or discontinuation.

From this perspective, management of luminal gas burden may represent a relevant component of supportive care during incretin therapy, particularly during periods of gastrointestinal adaptation.

### 5.3. Position of Simethicone Within the IMTA Framework

Within the proposed Incretin–Microbiota Tolerance Axis (IMTA), simethicone should not be regarded as a microbiota-modulating intervention. Instead, it occupies a distinct role within the luminal gas regulation domain, one of the three operational components of the framework.

While PHGG and SCFA-supportive approaches aim to influence microbial metabolic activity and intestinal homeostasis, simethicone addresses a downstream consequence of altered gastrointestinal transit, namely gas-related symptom burden. Its inclusion within IMTA therefore reflects a tolerability-oriented rather than a microbiota-oriented strategy.

This distinction is important because gastrointestinal tolerability itself represents a critical determinant of long-term treatment persistence. Even interventions that do not directly modify microbial composition may contribute to adherence if they reduce symptom burden during the early phases of therapy.

### 5.4. Current Evidence and Clinical Perspective

Direct evidence supporting simethicone use specifically in patients receiving GLP-1 receptor agonists or dual GIP/GLP-1 receptor agonists is currently lacking. Available evidence derives primarily from studies in functional bloating, dyspepsia, irritable bowel syndrome, and other gas-related gastrointestinal disorders, where simethicone has demonstrated a favorable safety profile and modest symptomatic benefits [17,18].

Accordingly, its use during incretin-based therapy should be regarded as a pragmatic, symptom-oriented supportive measure rather than an evidence-based adjunctive intervention. Within the proposed IMTA framework, simethicone may contribute to improving patient comfort during treatment initiation and dose escalation by reducing gas-related symptom burden, thereby potentially facilitating treatment persistence.

Future prospective studies should determine whether targeted management of luminal gas-related symptoms improves patient-reported outcomes, successful dose escalation, treatment persistence, and ultimately long-term cardiometabolic outcomes during incretin-based therapy.

## 6. Short-Chain Fatty Acids (SCFAs): The Cardiometabolic Bridge Between Gut Microbiota and Incretin Therapy

### 6.1. Biosynthesis, Composition, and Physiological Functions

Short-chain fatty acids (SCFAs)—primarily acetate, propionate, and butyrate—are generated through anaerobic microbial fermentation of non-digestible dietary substrates within the colon. SCFA production depends on multiple factors, including dietary fiber availability, intestinal transit time, microbial diversity, and the metabolic activity of the gut microbiota [7,14,19,20].

Among SCFAs, butyrate serves as the principal oxidative substrate for colonocytes and plays a central role in maintaining intestinal epithelial integrity. Propionate is primarily transported to the liver, where it may influence gluconeogenesis and lipid metabolism, whereas acetate enters the systemic circulation and contributes to peripheral energy homeostasis and neuroendocrine signaling [14].

Because incretin-based therapies substantially modify gastrointestinal transit dynamics and nutrient delivery, they may indirectly influence intestinal fermentation patterns and SCFA generation. These interactions suggest the existence of a functional relationship between incretin pharmacology and microbial metabolic activity within the gut environment.

### 6.2. The GLP-1–SCFA Positive Feedback Loop: A Mechanistic Keystone

One of the most relevant mechanistic interactions between SCFAs and incretin physiology involves stimulation of endogenous GLP-1 secretion by microbial metabolites. Experimental evidence indicates that butyrate and propionate may activate free fatty acid receptors FFAR2 (GPR43) and FFAR3 (GPR41) expressed on enteroendocrine L-cells, thereby promoting secretion of GLP-1 and peptide YY (PYY) [14,19,20].

This interaction suggests the presence of a bidirectional microbiota–incretin signaling loop in which dietary fiber fermentation contributes to endogenous incretin activity, while incretin-mediated alterations in motility and nutrient transit influence microbial fermentation patterns. Within this framework, preservation of SCFA-producing microbiota may theoretically support metabolic responsiveness and gastrointestinal adaptation during GLP-1 receptor agonist therapy.

Conversely, a dysbiotic intestinal environment characterized by reduced microbial diversity and impaired SCFA production may attenuate these adaptive mechanisms and potentially contribute to increased GI symptom burden. Although these concepts remain incompletely validated in clinical populations receiving incretin therapy, they provide a biologically plausible rationale for microbiota-supportive interventions during treatment.

### 6.3. Intestinal Barrier Integrity and Systemic Inflammation

SCFAs, particularly butyrate, contribute substantially to maintenance of epithelial barrier function and modulation of intestinal immune responses. Experimental studies demonstrate that butyrate may enhance expression of tight junction proteins, support mucus layer integrity, and reduce activation of pro-inflammatory signaling pathways, including nuclear factor kappa B (NF-κB) [7,16,19,21].

Through these mechanisms, SCFAs may help reduce intestinal permeability and limit systemic translocation of lipopolysaccharide (LPS), a process implicated in chronic low-grade inflammation, insulin resistance, and cardiometabolic disease progression [7,16,19,21]. In addition, SCFAs appear to influence regulatory T-cell activity and mucosal immune homeostasis, further supporting their potential role in maintaining intestinal eubiosis.

These observations have generated growing interest in the gut–heart axis as a potential therapeutic target in obesity and metabolic disease. Although direct evidence linking SCFA modulation to cardiovascular outcomes during incretin therapy remains limited, preservation of intestinal barrier integrity may represent an important supportive mechanism within broader cardiometabolic risk reduction strategies.

### 6.4. SCFAs and Cardiometabolic Outcomes

Observational and mechanistic studies have reported associations between higher SCFA availability and favorable metabolic parameters, including reduced visceral adiposity, improved insulin sensitivity, lower triglyceride concentrations, and reduced inflammatory marker expression [7,14,19,20,22]. Experimental studies have also suggested potential effects of propionate on hepatic lipid metabolism and appetite regulation.

These mechanisms overlap partially with pathways implicated in the cardiometabolic benefits observed during GLP-1 receptor agonist therapy. Accordingly, SCFA-supportive interventions may theoretically complement incretin-based treatment through modulation of intestinal inflammation, metabolic signaling, and epithelial barrier function.

However, direct clinical evidence demonstrating additive cardiovascular benefit from SCFA-targeted strategies during GLP-1 therapy is currently insufficient. Future studies incorporating microbiome profiling, SCFA quantification, and cardiometabolic outcome assessment will be necessary to clarify the clinical significance of these interactions.

**Figure 2:**
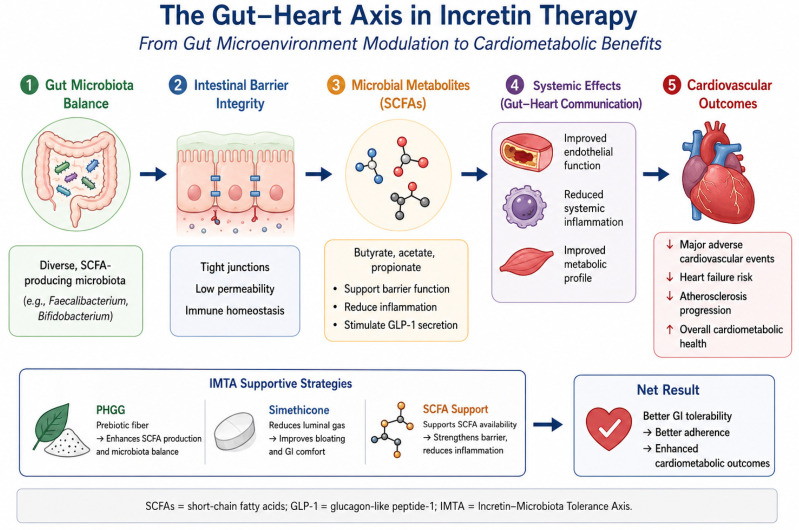
The gut–heart axis in incretin-based therapy. Proposed interactions between gut microbiota, SCFA production, intestinal barrier function, systemic inflammation, and cardiovascular health. Gut-directed supportive strategies may contribute to preservation of gastrointestinal tolerability and long-term cardiometabolic benefit during incretin-based treatment.

To illustrate the proposed mechanistic links between microbial metabolites, intestinal barrier integrity, systemic inflammation, and cardiovascular outcomes, Figure 2 summarizes the gut–heart axis in the context of incretin-based therapy.

### 6.5. Strategies to Support SCFA Production During Incretin Therapy

Several nutritional and microbiota-supportive approaches may help sustain SCFA production during incretin-based treatment (Table 1).

Because gastrointestinal tolerability varies substantially among individuals receiving incretin therapy, SCFA-supportive strategies should ideally be individualized according to symptom profile, dietary habits, and baseline gastrointestinal sensitivity.

At present, most evidence supporting these interventions remains mechanistic or extrapolated from non-GLP-1 populations. Prospective randomized studies are required to determine whether SCFA-targeted approaches can improve GI tolerability, treatment adherence, or long-term cardiometabolic outcomes during incretin-based pharmacotherapy.

This table summarizes nutritional and microbiota-supportive strategies potentially capable of enhancing SCFA production and preserving intestinal homeostasis during incretin-based pharmacotherapy. The listed interventions may contribute to maintenance of epithelial barrier integrity, modulation of microbial fermentation dynamics, and improvement of gastrointestinal tolerability during GLP-1 receptor agonist and dual GIP/GLP-1 receptor agonist treatment. None of the listed interventions should be interpreted as evidence-based therapeutic recommendations for routine clinical practice. Current evidence remains primarily mechanistic or extrapolated from metabolic and gastrointestinal research outside dedicated incretin-treated populations.

## 7. The Incretin–Microbiota Tolerance Axis (IMTA): Clinical Framework and Implementation

### 7.1. Conceptual Architecture

The Incretin–Microbiota Tolerance Axis (IMTA) is proposed as a conceptual framework integrating gastrointestinal tolerability, microbial metabolic function, and long-term adherence during incretin-based therapy. Within this model, gastrointestinal adverse events are viewed not solely as isolated pharmacological side effects, but also as manifestations of altered interactions between intestinal motility, luminal fermentation dynamics, microbial composition, and epithelial barrier integrity.

The IMTA framework is organized around three interconnected operational domains:

1. Motility homeostasis—GLP-1 receptor agonists and dual GIP/GLP-1 receptor agonists substantially alter gastric emptying and intestinal transit. Supportive strategies aimed at maintaining balanced gastrointestinal motility may help reduce constipation, dyspeptic symptoms, and fermentation-related discomfort.
2. Luminal gas regulation—Altered transit dynamics and proximal fermentation may contribute to excessive intraluminal gas accumulation during dose escalation. Targeted gas-management approaches, including simethicone administration, may improve symptomatic tolerability during treatment initiation.
3. Microbial metabolic competence—Preservation of short-chain fatty acid (SCFA)-producing microbiota may support epithelial barrier integrity, intestinal immune homeostasis, and endogenous incretin signaling. Nutritional and prebiotic strategies, including PHGG supplementation and Mediterranean dietary patterns, may contribute to maintenance of microbial metabolic activity during therapy.

These domains are biologically interconnected. Altered motility may influence microbial fermentation patterns, while microbial metabolites such as SCFAs may in turn affect intestinal barrier function, inflammatory signaling, and neuroendocrine regulation. Within this framework, gastrointestinal tolerability and microbial homeostasis are viewed as potentially complementary determinants of treatment persistence and long-term cardiometabolic benefit.

### 7.2. Phase-Based Clinical Integration

A phase-oriented supportive strategy may facilitate gastrointestinal adaptation during incretin therapy initiation and dose escalation (Table 2). This phased approach is intended as a practical and hypothesis-generating framework rather than a validated treatment protocol. Because GI symptom burden varies considerably among individuals, supportive interventions should be individualized according to baseline gastrointestinal function, dietary habits, symptom severity, and treatment response.

The proposed framework is intended to complement, rather than replace, current evidence-based supportive care, which includes individualized dose titration, nutritional assessment, adequate hydration, sufficient protein intake, resistance exercise, symptom-directed pharmacological management, and individualized dietary counseling, as recommended by recent multidisciplinary nutritional guidance and contemporary obesity management algorithms [23,24].

The IMTA concept specifically explores whether optimization of the intestinal microenvironment may represent an additional supportive strategy beyond these established measures.

The following framework is intended solely to illustrate a hypothesis-generating conceptual application of the IMTA framework. It should not be interpreted as a validated clinical management protocol or as evidence-based guidance for the routine management of patients receiving incretin-based therapy.

This table summarizes a proposed phase-oriented supportive approach aimed at improving gastrointestinal tolerability and preserving intestinal homeostasis during incretin-based pharmacotherapy. The IMTA framework integrates strategies targeting gastrointestinal motility, luminal gas management, and microbial metabolic support across different stages of GLP-1 receptor agonist and dual GIP/GLP-1 receptor agonist treatment. The proposed interventions are mechanistically based and hypothesis-generating, and should not be interpreted as evidence-based therapeutic recommendations pending prospective validation studies.

### 7.3. Clinical Situations Requiring Individualized Management

Although the proposed IMTA framework may support gastrointestinal adaptation in selected patients, it should not delay recognition of clinically significant gastrointestinal complications requiring individualized evaluation or modification of therapy. Patients presenting with persistent vomiting, severe constipation, symptoms suggestive of gastroparesis or bowel obstruction, clinically relevant dehydration, or progressive nutritional compromise require prompt clinical reassessment. In such situations, optimization of supportive strategies should be accompanied by consideration of dose reduction, temporary treatment interruption, or specialist gastroenterological evaluation when appropriate.

Similarly, because fermentable fibers may transiently increase gas production and abdominal bloating during early supplementation, microbiota-supportive interventions should be introduced cautiously and individualized according to baseline gastrointestinal function and symptom burden. Nutritional assessment, adequate hydration, sufficient protein intake, and preservation of lean body mass through resistance exercise remain essential components of comprehensive supportive care throughout incretin-based therapy [22,23,24]. Consequently, the IMTA framework should be regarded as a complementary supportive approach rather than a substitute for established clinical management of gastrointestinal adverse events.

### 7.4. Treatment Adherence and Population-Level Implications

Long-term adherence remains one of the principal determinants of clinical effectiveness during incretin-based therapy. Although incretin-based treatments demonstrate substantial efficacy under controlled trial conditions, gastrointestinal adverse events continue to represent a major barrier to sustained use in routine clinical practice. Importantly, treatment persistence is influenced by multiple determinants extending beyond gastrointestinal tolerability. The IMTA framework specifically addresses gastrointestinal intolerance as one potentially modifiable component of treatment persistence and should not be interpreted as a comprehensive explanation for treatment discontinuation, which is also affected by socioeconomic factors, healthcare access, medication availability, patient preference, and therapeutic expectations.

From a population-health perspective, even modest improvements in treatment persistence could potentially translate into meaningful preservation of metabolic and cardiovascular benefit over time. Strategies capable of reducing early discontinuation may therefore have implications extending beyond symptomatic management alone.

However, no randomized controlled studies have directly evaluated whether integrated microbiota-supportive approaches—including PHGG supplementation, luminal gas management, and SCFA-targeted interventions—improve adherence or cardiometabolic outcomes in patients receiving incretin-based therapy. Consequently, the IMTA framework should currently be regarded as mechanistically grounded but preliminary, requiring prospective clinical validation.

### 7.5. The Gut–Heart Axis as a Therapeutic Target

Increasing evidence supports the existence of bidirectional communication between the gut microbiota, intestinal immune system, and cardiovascular physiology, often referred to as the “gut–heart axis” [6,9,14,17]. SCFAs may influence systemic inflammation, endothelial function, hepatic lipid metabolism, and vascular signaling pathways implicated in cardiometabolic disease progression.

Because many of these pathways overlap mechanistically with the therapeutic targets of incretin-based therapies, optimization of the intestinal microenvironment may theoretically complement established cardiometabolic therapies. In this context, gut-directed supportive strategies could potentially represent an adjunctive component of integrated obesity and cardiovascular risk management.

Nonetheless, direct evidence linking microbiota modulation during incretin therapy to improved cardiovascular outcomes remains limited. Future translational and clinical studies incorporating microbiome analysis, inflammatory biomarkers, SCFA quantification, and cardiovascular endpoints will be essential to clarify the clinical relevance of the proposed IMTA model. More broadly, recent reviews have reinforced the concept that gut microbiota–host interactions influence systemic disease through mechanisms involving immune regulation, epithelial barrier function, and microbial metabolites, although these observations have not been specifically validated in the context of incretin-based therapy [25].

## 8. Implications for Clinical Practice and Future Research

### 8.1. Clinical Phenotypes Most Likely to Benefit from IMTA-Based Supportive Strategies

Although the proposed Incretin–Microbiota Tolerance Axis (IMTA) framework may have broad applicability during incretin-based therapy, certain patient populations may be particularly susceptible to gastrointestinal intolerance and impaired treatment persistence.

These include:

1. Patients with pre-existing functional gastrointestinal disorders, including irritable bowel syndrome, functional dyspepsia, or chronic bloating, in whom incretin-induced alterations in gastrointestinal motility may exacerbate baseline symptom burden.
2. Patients with features suggestive of intestinal dysbiosis, including low dietary fiber intake, prior frequent antibiotic exposure, obesity-associated metabolic dysfunction, or reduced microbial diversity.
3. Patients with type 2 diabetes and autonomic dysfunction, particularly those with delayed gastric emptying or diabetic gastroparesis, in whom GLP-1-mediated slowing of gastric transit may be more clinically significant.
4. Patients receiving GLP-1 receptor agonists for cardiovascular risk reduction, especially individuals meeting criteria similar to those enrolled in the SELECT trial [3], where long-term treatment persistence may be critical for maintaining cardiometabolic benefit.
5. Patients undergoing higher-dose incretin therapy, including semaglutide 2.4 mg weekly or tirzepatide 15 mg weekly, who may experience greater gastrointestinal symptom burden during dose escalation [1,2].

Recognition of these clinical phenotypes may help clinicians identify individuals who could benefit from earlier supportive intervention targeting gastrointestinal tolerability and microbial homeostasis.

### 8.2. Nutritional Support Beyond the Gut Microenvironment

Although the present review focuses primarily on modulation of the intestinal microenvironment, supportive care during incretin-based therapy should also address preservation of overall nutritional status and body composition. Rapid weight loss induced by GLP-1 receptor agonists and dual GIP/GLP-1 receptor agonists is accompanied by reductions in both fat mass and lean body mass, with the relative contribution of lean tissue varying across studies and therapeutic agents [26].

Adequate dietary protein intake, resistance exercise, and individualized nutritional counseling therefore remain essential components of comprehensive supportive care throughout incretin therapy [23,24]. These interventions may help preserve skeletal muscle mass and physical function while optimizing long-term metabolic health.

Accordingly, the proposed IMTA framework should be viewed as complementary to established nutritional and lifestyle recommendations rather than as a substitute for comprehensive obesity management. Future studies should evaluate whether combining microbiota-supportive strategies with interventions aimed at preserving lean body mass further improves treatment persistence and long-term cardiometabolic outcomes.

### 8.3. Research Priorities

The IMTA framework generates several clinically relevant and testable research questions that warrant prospective investigation.

Potential areas for future study include:

Randomized controlled trials evaluating PHGG and simethicone during incretin-based therapy, with assessment of gastrointestinal symptom burden, treatment persistence, and dose-escalation completion rates.

Mechanistic microbiome studies examining changes in microbial composition, SCFA production, and intestinal permeability markers during incretin therapy with or without microbiota-supportive interventions.

Biomarker-based prediction models evaluating whether baseline microbiome characteristics, fecal SCFA concentrations, or markers of epithelial barrier dysfunction can predict gastrointestinal tolerability and treatment discontinuation risk.

Translational cardiovascular investigations assessing whether modulation of the intestinal microenvironment influences inflammatory biomarkers, endothelial function, or surrogate cardiovascular endpoints during incretin-based pharmacotherapy.

Precision nutrition approaches integrating dietary fiber profiling, microbiome assessment, and individualized gastrointestinal symptom management during long-term obesity treatment.

Importantly, future studies should distinguish between mechanistic plausibility and clinically meaningful outcome improvement. While current evidence supports a biologically coherent rationale linking gut microbial function and incretin tolerability, direct interventional evidence remains limited.

### 8.4. Toward Precision Cardiometabolic Pharmacotherapy

The growing integration of microbiome science into metabolic medicine suggests that intestinal physiology may become increasingly relevant within future precision cardiometabolic care models. Just as pharmacogenomics and individualized cardiovascular risk stratification are progressively informing therapeutic decision-making, microbiota-related factors may eventually contribute to personalization of incretin-based treatment strategies.

Within this evolving framework, assessment of gastrointestinal vulnerability, dietary patterns, microbial metabolic activity, and intestinal barrier function could potentially help identify patients at increased risk for GI intolerance or treatment discontinuation.

At present, however, microbiome-guided incretin therapy remains investigational. The supportive interventions discussed in this review—including PHGG supplementation, luminal gas management, and SCFA-supportive dietary approaches—should therefore be viewed as pragmatic, low-risk, and mechanistically grounded adjunctive strategies rather than evidence-based standards of care.

Ultimately, the IMTA framework proposes that optimization of the intestinal microenvironment may represent a clinically relevant supportive component of long-term incretin therapy, particularly in patients at high risk for gastrointestinal intolerance and impaired adherence.

## 9. Limitations

This narrative review has several limitations that should be acknowledged. First, much of the evidence supporting the proposed Incretin–Microbiota Tolerance Axis (IMTA) framework is mechanistic, extrapolative, or derived from studies conducted in populations outside dedicated incretin-treated cohorts. Although partially hydrolyzed guar gum (PHGG), simethicone, and short-chain fatty acids (SCFAs) each demonstrate biological plausibility and supportive evidence in gastrointestinal and metabolic research, direct randomized controlled trials evaluating these interventions during GLP-1 receptor agonist or dual GIP/GLP-1 receptor agonist therapy are currently lacking.

Second, the relationship between gut microbiota composition, gastrointestinal tolerability, and long-term cardiometabolic outcomes during incretin therapy remains incompletely understood. The intestinal microbiome is characterized by substantial interindividual variability influenced by diet, pharmacological exposure, metabolic status, genetics, and environmental factors, limiting the generalizability of microbiota-targeted strategies across heterogeneous patient populations. Furthermore, the proposed IMTA framework specifically focuses on gastrointestinal tolerability and does not encompass the multiple non-gastrointestinal determinants of treatment persistence, including healthcare access, socioeconomic factors, medication availability, patient preference, and perceived treatment effectiveness. Accordingly, the framework should be interpreted as addressing one potentially modifiable contributor to long-term treatment persistence rather than a comprehensive explanation for treatment discontinuation.

Third, several mechanistic pathways discussed in this review—including the proposed interaction between SCFA production, endogenous incretin signaling, and cardiovascular benefit—remain hypothesis-generating and have not yet been validated in prospective human studies specifically designed to assess these interactions during incretin-based pharmacotherapy. Furthermore, no human study has yet validated the complete sequence linking incretin therapy, microbiota alteration, gastrointestinal intolerance, treatment discontinuation, and loss of cardiometabolic benefit. Accordingly, the IMTA should be interpreted as a conceptual framework integrating biologically plausible but independently supported mechanisms, rather than as a proven causal pathway.

In addition, much of the currently available incretin outcome literature originates from large industry-sponsored clinical trials primarily focused on weight reduction, glycemic control, and cardiovascular endpoints rather than gastrointestinal physiology or microbiome-related outcomes. Consequently, dedicated data addressing microbial composition, intestinal permeability, fermentation dynamics, and GI adaptation during long-term incretin therapy remain limited.

Finally, although the IMTA framework is intended as a clinically oriented and mechanistically grounded model, it should not be interpreted as an evidence-based therapeutic protocol. The supportive strategies discussed in this review should currently be considered exploratory adjunctive approaches requiring prospective validation before routine implementation within standardized clinical pathways.

## 10. Conclusions

Glucagon-like peptide-1 receptor agonists and dual GIP/GLP-1 receptor agonists represent some of the most effective pharmacological therapies currently available for obesity and cardiometabolic disease management. In addition to promoting substantial weight reduction and glycemic improvement, these agents have demonstrated clinically meaningful cardiovascular benefit in high-risk populations, reinforcing their growing role within long-term cardiometabolic care [1,2,3].

Despite these advances, gastrointestinal adverse events remain a major limitation to sustained treatment adherence in routine clinical practice. Nausea, bloating, constipation, altered bowel habits, and early satiety frequently contribute to dose reduction or premature treatment discontinuation, potentially limiting the long-term metabolic and cardiovascular benefits associated with continued incretin therapy.

This review proposes that the intestinal microenvironment may represent one of several potential modifiers, although incompletely characterized, determinant of gastrointestinal tolerability during incretin-based pharmacotherapy. Within the proposed Incretin–Microbiota Tolerance Axis (IMTA) framework, supportive strategies targeting intestinal motility, luminal gas dynamics, microbial fermentation, and short-chain fatty acid (SCFA) production may help preserve gastrointestinal homeostasis and facilitate long-term treatment persistence.

Partially hydrolyzed guar gum (PHGG) may contribute to motility regulation and support SCFA-producing microbiota, while simethicone may provide targeted symptomatic relief from luminal gas-related discomfort during dose escalation. SCFA-supportive interventions may additionally contribute to maintenance of epithelial barrier integrity and modulation of inflammatory pathways relevant to metabolic disease.

Importantly, many of the concepts discussed in this review remain mechanistically based and hypothesis-generating. At present, no human study has validated the complete sequence linking incretin therapy, microbiota alteration, gastrointestinal intolerance, treatment discontinuation, and loss of long-term cardiometabolic benefit. Consequently, the proposed IMTA should be regarded as a conceptual framework intended to stimulate future translational and clinical research rather than as an established mechanistic model.

Prospective randomized studies incorporating microbiome profiling, SCFA quantification, gastrointestinal symptom assessment, and cardiometabolic outcome evaluation will be necessary to determine the clinical validity and therapeutic relevance of microbiota-supportive approaches during incretin therapy.

Pending such evidence, microbiota-supportive interventions should currently be regarded as hypothesis-generating supportive approaches that require prospective clinical validation before routine implementation in patients receiving incretin-based therapy.

## Figures and Tables

**Table 1:** Candidate microbiota-supportive strategies potentially applicable during incretin therapy.

Strategy	Primary Mechanism	Clinical Application
PHGG (investigational microbiota-supportive strategy)	Selective promotion of SCFA-producing microbiota	Support GI tolerability and microbial homeostasis
Resistant starch RS2/RS3	Colonic butyrate generation	Support fermentation diversity and metabolic signaling
Butyrate supplementation (investigational)	Direct SCFA supplementation	Experimental microbiota-supportive strategy requiring clinical validation
Inulin/FOS	Prebiotic stimulation of microbial fermentation	Support SCFA generation as a microbiota-supportive strategy
Mediterranean dietary pattern	Increased fiber and polyphenol diversity	Promote microbiota richness and anti-inflammatory activity

SCFA = short-chain fatty acid, PHGG = partially hydrolyzed guar gum, RS2 = resistant starch type 2, RS3 = resistant starch type 3, FOS = fructooligosaccharides, GLP-1 = glucagon-like peptide-1, GI = gastrointestinal.

**Table 2:** Hypothesis-generating phase-oriented framework for future evaluation for the Incretin–Microbiota Tolerance Axis (IMTA) during incretin-based therapy.

Phase	Primary GI Challenge	IMTA Adjunct Strategy	Therapeutic Target
Week 1–4 (Initiation)	Nausea, bloating, early satiety	Consideration of symptom-directed gas management and PHGG supplementation	Improve early GI tolerability and reduce premature discontinuation
Week 4–8 (Titration)	Constipation, altered bowel habits, microbial imbalance	Consideration of microbiota-supportive fiber strategies	Support motility homeostasis and microbial adaptation
Week 8+ (Maintenance)	Chronic dysbiosis risk, adherence decline	Mediterranean dietary pattern and individualized fiber optimization	Sustain adherence and metabolic stability

IMTA = Incretin–Microbiota Tolerance Axis, GLP-1 = glucagon-like peptide-1, GI = gastrointestinal, PHGG = partially hydrolyzed guar gum, SCFA = short-chain fatty acid.

## Data Availability

The original contributions presented in the study are included in the article; further inquiries can be directed to the corresponding author.
